# Scanning Mortars to Understand the Past and Plan the Future for the Maintenance of Monuments

**DOI:** 10.1155/2018/7838502

**Published:** 2018-07-18

**Authors:** Maria Stefanidou, Eleni Pavlidou

**Affiliations:** ^1^Laboratory of Building Materials, School of Civil Engineering, Aristotle University of Thessaloniki, Thessaloniki, Greece; ^2^Solid State Section, Physics Department, Aristotle University of Thessaloniki, Thessaloniki, Greece

## Abstract

The effect of microstructure on macroproperties of building materials was the initiation in order to use microscopic techniques for studying the materials' behavior. Primer role among the different techniques has the scanning electron microscope (SEM) as it provides much information in an easy and understandable way. SEM has been used in almost every study of the last decades, dealing with historic and repair materials to complete the analysis performed. In the case of historic mortars, it is a unique technique as it requires a small representative sample and without any intense treatment important information derived from an almost unknown sample including damage detection, phase identification, and microanalysis. It is usually a complementary method of analysis but a precious one as the gained results from the analysis of old mortars are used for designing compatible repair materials for restoration purposes. In the paper, the long-term use of SEM in studying both old authentic and innovative repair mortars is presented.

## 1. Introduction

Microscopic analysis of material's structure is valuable for many applications, such as the material's development and improvement, quality control, reverse engineering, and evaluation of performance [1]. The complexity of materials' microstructure makes the constitution of a realistic model very difficult for the prediction of their behavior [2]. For that reason, microscopy techniques for the investigation of materials have been used since the decade of 40's [3]. The analysis and understanding of microstructure have played a vital role in understanding the old and in planning repair materials in a compatible and durable way. The term microscopy is referring to the techniques that the samples can be viewed at an appropriate scale for examination. In scanning electron microscopy (SEM), the image is formed by electronic processing of the wave nature. It is considered, among the most used, fast and accurate method of analysis for the examination of building materials, which are used for construction purposes. SEM has been extensively used for the material characterization, especially in combination with energy dispersive X-ray microanalysis (SEM/EDS) [4]. This technique covers a wide range of magnification, roughly from 10x to 300,000x. The electron beam scans the specimen's surface, forming the image with an appropriate scale. The most important advantages are the ease of magnification change and the large depth of focus. When scanning a specimen's surface with a finely focused electron beam, secondary electrons, backscattered electrons, and X-rays are emitted, carrying information about the sample. The image displayed is dependent on the acquisition and processing of signals, produced from the interaction of the electron beam with the specimen. These interactions can be broken down into two major categories, those resulting in elastic collisions of the electron beam on the sample and those resulting in inelastic collisions, where kinetic energy is not conserved throughout the encounter. The most widely utilized signal produced by the interaction of the primary electron beam with the sample is the secondary electron emission signal. Secondary electrons are characterized from other electrons by having energy less than 50 eV. This image is the most useful for examining surface structure and provides the best resolution image among the various scanning signals. Depending on the initial size of the primary beam and various other conditions (composition of sample, accelerating voltage, and position of specimen relative to the detector), a secondary electron signal can resolve surface structures down to the order of 10 nm. The topographical image is dependent on how many of the secondary electrons actually reach the detector.

A second type of electrons, the backscattered electrons, is also produced when the specimen is irradiated with the primary electron beam. Together, backscattered and secondary electrons contribute to the signal that reaches the scintillator and form what we refer to as the secondary electron image. A backscatter electron is defined as one which has undergone a single or multiple scattering events and escapes with an energy greater than 50 eV. Backscattered electrons are produced as the result of elastic collisions with the atoms of the sample and usually retain about 80% of their original energy. The number of backscattered electrons produced increases with increasing atomic number of the specimen. The region of the specimen from which backscattered electrons are produced is considerably larger than that for secondary electrons, and the resolution of a backscattered electron image is considerably less (1 *μ*m) than that for a secondary electron image (10 nm). Because of their greater energy, backscattered electrons can escape from much deeper regions of the sample than can secondary electrons, hence the larger region of excitation. The formation of the images depends upon the acquisition time selected and varies from a few to several minutes.

Another class of signals produced by the interaction of the primary electron beam with the specimen comes under the category of characteristic X-rays. When an electron from an inner atomic shell is displaced by colliding with a primary electron, it leaves a vacancy in that electron shell. In order to reestablish the proper balance in its orbitals following an ionization event, an electron from an outer shell of the atom may “fall” into the inner shell and replace the “hole” vacated by the displaced electron. In doing so, this falling electron loses energy and this energy is emitted and referred to as X-radiation or X-rays. The SEM can be set up in such a way that the characteristic X-rays of a given element are detected with an energy dispersive X-ray spectrometer (EDS). By analyzing the characteristic X-rays generated, the elements that constitute the specimen can be identified and this creates the qualitative and quantitative elemental analysis of the surface detected. Additionally, their position can be recorded or “mapped.” These X-ray maps can be used to form an image of the sample that shows where atoms of a given element are localized. The resolution of these X-ray maps is on the order of greater than 1 *μ*m. In this way, specific desired characteristics of a specimen's surface can be seen on the monitor in a magnified scale.

Scanning electron microscopy requires electrically conductive specimens. Insulating specimens need to be covered by a thin (1–10 nm) conducting surface layer, usually applied by sputtering with gold in an argon atmosphere, or with carbon coating in vacuum. Sample size can be a maximum of 10 cm in diameter and 50 mm in height, but there is a restriction in the specimen's movement, so the area of interest should be about 2.5 cm in diameter around the center of the goniometer stage. Restrictions also apply regarding the maximum height, tilt, and rotation, depending on the specific stage.

The above characteristics make SEM with X-ray microanalysis very effective in morphology investigation, as easily produced micrographs provide topographical information together with the qualitative and quantitative analysis, evaluating the characteristics of the materials. The SEM used for this study is a JSM 840 A, assisted by OXFORD INCA EDS analysis and applied both on old historic mortars in order to understand their nature as well as on repair mortars in order to record the achieved properties.

## 2. Study of Old Mortars

**Figure 1:**
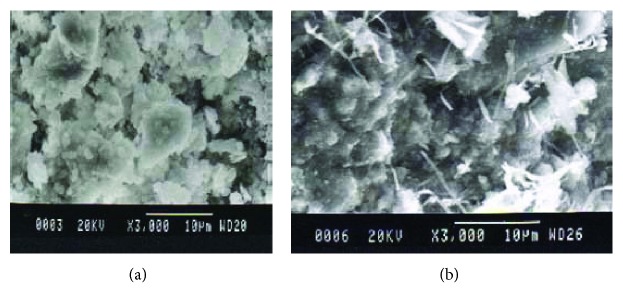
Lime mortar from Bezesteni (a) with calcite plates and pozzolanic mortar from Hagia Sophia (Thessaloniki) (b) with fibrous crystals of C-S-H composition.

The abovementioned characteristics of the SEM can be utilized in order to analyze the heterogeneous mortars' structure. The study of an old mortar, taken from a monument or historic building, is usually a “black box” as no data can be obtained from any source (except the rare cases where information can be taken from archaeological work). Approaching the physical, mechanical, chemical, and mineralogical properties of old mortar, aiming to decode its behavior into the structure, requires combined techniques (usually costly, time-consuming, and needing a significant quantity of material) [5, 6]. To understand the components and their proportion into the mortar mixture is a key factor in order to understand the behavior of old mortars [7]. From the analysis performed so far, it has been proved that they were based on lime, and when it was necessary, binders were combined [8]. The use of natural pozzolan and brick dust with lime was a common technique, in cases where special needs were covered, such as high strength and water tightness [9]. The microstructure analysis of old mortars reveals the different behaviors of pure lime and pozzolanic mortars. From Figure 1(a), the loose cohesion of calcite plates is recorded in pure lime mortar taken from Bezesteni (15th century). Also, pores and open spaces are present in the structure. In the pozzolanic mortar from Hagia Sophia in Thessaloniki (6th century AD), a compact structure with very small pores and fibrous crystals of C-S-H composition is recorded (Figure 1(b)). The role of binders in old mortars is of paramount importance for the quality and durability of the mortar [10, 11]. An important parameter of the quality of old mortars is the size, chemical composition, and how the crystals of the binder are bounded together. A stable connective fine-crystallized tissue is created that contributes to coherent binder. The technique has also been used by researchers and archaeologists, investigating the production technology of old mortars and the socioeconomic aspect of the provenance of their raw material, as in the case of prehistoric mortars in Cyprus. These provide strong evidence of early intentional use of artificial pozzolanic materials in lime mortars, which can also significantly contribute to the comprehension of the pathway of knowledge transfer to other civilizations [12].

**Figure 2:**
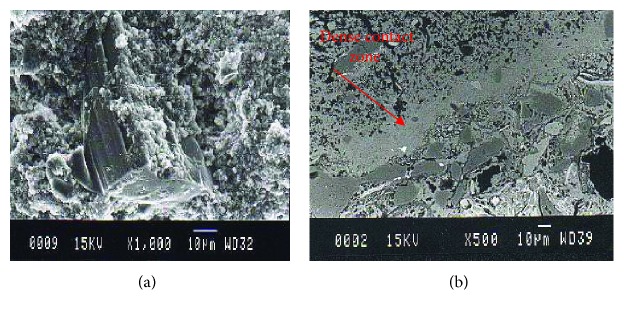
Contact of pozzolanic paste with natural aggregate (a) and strong cohesion between a brick fragment and a pozzolanic binder from Galerious Palace (4th century AD) (b).

The aggregates used in old mortars were usually natural in origin, while crushed ceramic or limestone pebbles in various granulometries with even distribution were also used in some cases [13, 14]. The binder-aggregate transition zone was recorded strong in some cases where brick aggregates were recorded in combination with pozzolanic binder while in cases of natural aggregates, a loose mechanical bond was recorded (Figure 2). The proportion of aggregates in relation to the binder, the maximum size used, and their gradation seem to directly affect the volume stability and the form of discontinuities such as pores and cracks [15]. In order to assess the role of the aggregate type in the properties of air lime mortar, scholars have used microanalysis to record differences at the binder/aggregate interface [16].

**Figure 3:**
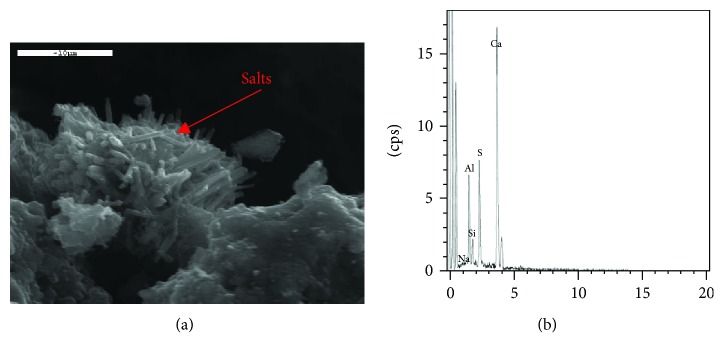
(a) Salt crystals in the structure of hydraulic mortars. (b) X-ray analysis on the crystals of (a).

Because of long exposure, old mortars develop different pathology symptoms such as cracks, crumbling, and erosion [17]. To study the ageing of various materials as they degrade with time, it is important to analyze the microstructure changes supplemented with measurement of the area of damages in the form of microvoids [18]. SEM micrographs reveal loose microstructures in damaged materials and uniform and dense microstructures in durable materials. Damage in porous materials due to salts is a common phenomenon and occurs to old and new repair materials (Figure 3). There are various forms of salt decay, such as cracks, scaling, efflorescence, crypto-florescence, pulverization, and material loss [19]. Old mortars present an admirable durability and longevity, despite the fact that they were subjected to many wetting-drying cycles and to extreme conditions. According to studies, the crystallization of salts takes place when salts exert internal pressures, which exceed the strength of the material [20]. The size of the salt crystals seems to be correlated to the porous nature of the mortar paste. In porous lime mortars, crystals are formed into the spacious paste and pores and cracks remain empty. On the contrary, in hydraulic mortars, where the paste is dense, salt crystals may be also formed in small cracks [21]. On the basis of the damage mechanisms encountered in mortars, in cases where swelling of mortars is observed due to the mechanism of thaumasite formation, SEM has proved indicative [22]. The presence of this salt was documented, and the team proposed measures to avoid it.

**Figure 4:**
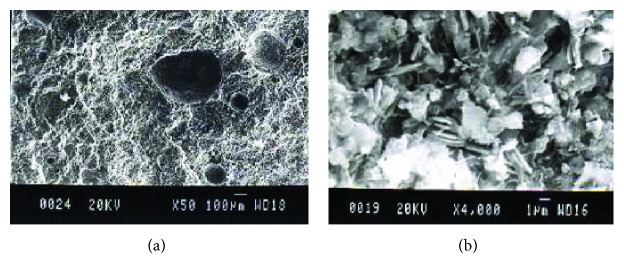
(a) Pores of diameter 50–250 *μ*m in the binder (SEM ×50). (b) Micropores of 700–1300 nm between crystal units (SEM ×4,000).

SEM is also used for the study of porosity since it has high resolution limit allowing a wide range of pore sizes to be recorded and the geometry can exactly be described (Figure 4).

By measuring, the area of voids from micrographs taken from historical mortars, qualitative and quantitative results of the damaged area, and the materials used can be estimated. Also, the porosity influences the macroscopic properties of the mortars and it is strongly correlated with the material's decay process. The uniform pore distribution recorded in old mortars allows some flow of water inside the structure without being held there. The porosity that characterizes old mortars (20–40%) is mainly due to micropores of 0.1–1 *μ*m [23]. Cracks have also been observed usually in the binder-aggregate transition zone, as a consequence of the poor cooperation between the two phases or within the binder due to shrinkage phenomena. The coexistence of pores and cracks, which is the most common case, makes the structure vulnerable, especially when cracks join the pores, forming “interconnecting phases” within the structure. This is more critical when these openings have access to the surface, consisting of paths through which deteriorating agents penetrate and settle inside the material's structure. The geometric characteristics, the position, the way of communication, and the crack orientation are factors affecting the strength. Pores may be isolated or filled with secondary crystals (salts, calcite). Large pores are limited to well-compacted mortars.

**Figure 5:**
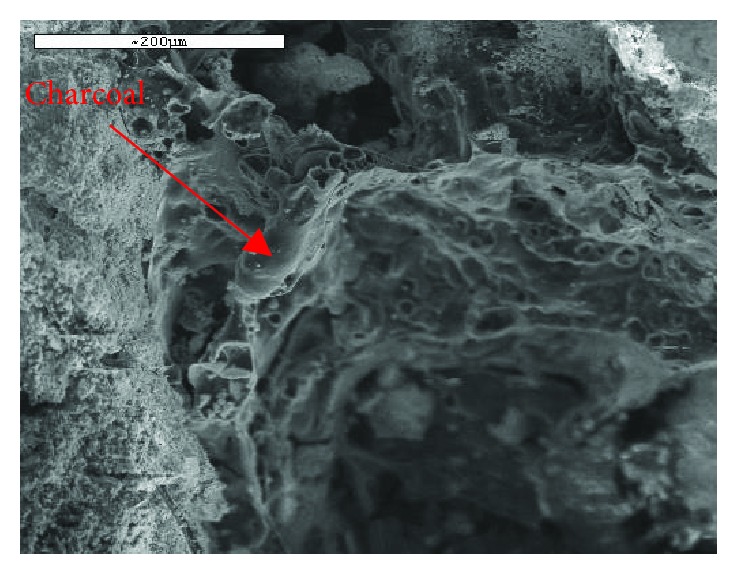
SEM analysis in charcoal of a Roman mortar from Galerius Palace (4th century AD).

The microscopic study of ancient mortars reveals that apart from the main ingredients, binders and aggregates, there are other materials as well, in small percentages in the mass, such as shells, charcoal particles, lime lumps, chips of wood, or straw (Figure 5). These enclosures are met in structural mortars as well as in renders, and they are observed in different historical periods (from the mud mortars of archaic period to strong pozzolanic mortars of Roman and Byzantine era and even in the mortars of 19th century or precement period) [24]. Their role in the behavior of ancient mortars, as well as their origin, is not always clear, but their morphologic characteristics (shape, size, and distribution), their percentage (not more than 2 wt %), and their cohesion with the binder (usually strong) give useful information about the older technology of building construction.

SEM and EDS analysis of mortars indicated the presence of organic fibers and calcite, quartz, and muscovite minerals of Ottoman mortars [25]. This research team identified the hydration-dehydration products by the morphological examination and microanalysis and the moderately uniform distribution of the porosity observed, which was attributed to the homogeneous distribution of the hydration products.

**Figure 6:**
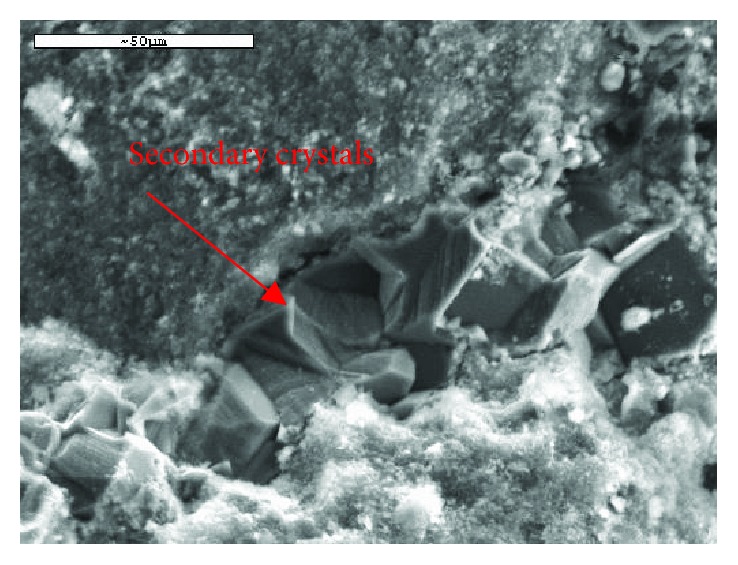
Detail of the recrystallization in the transition zone.

Secondary recrystallization phases are often observed in the structure of ancient lime-based mortars. They are mainly situated in previously formed cracks, in spherical pores, and in the loose transition zone (Figure 6). The composition of these phases depends mainly on the type of binders used and the hydrothermal conditions to which the mortar was subjected during its service life. The secondary crystals reduce the porosity, as they fully or partly cover pores and cracks [26].

SEM analysis performed in historic mortars reveals that the most durable mortars combine natural and brick aggregates with smooth granulometry, in the presence of few coarse grains and low proportion of fine aggregates in combination with strong binders. In the old mortars, the high proportion of aggregates is not always a quality criterion. Application of different aggregate/binder ratios, depending on the location of the mortar in the structure, the type of binder, and the construction environment, as applied by the old craftsmen, seems to have had many positive results in durability and quality of mortars. The combination of higher-quality lime with reactive natural pozzolan formed a strong binding system consisting of micrograined well-connected crystals.

## 3. Study of Repair Mortars

**Figure 7:**
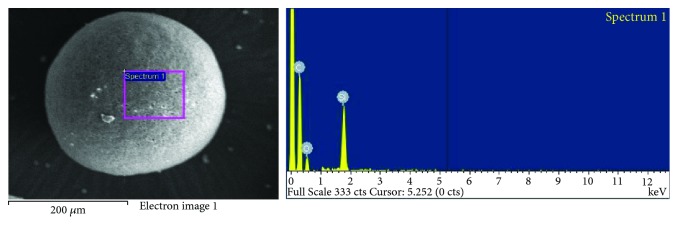
Research on the use of phase change materials as raw material in order to improve the thermal behavior of traditional lime mortar.

In order to design new repair mortars, many parameters are taken into account, such as the properties of old mortars, the use of proper available materials, the structure itself, and its environment [27]. Principles of compatibility have to be in equilibrium with durability aspects in terms of efficient and economic restoration, which will protect the old structure without causing side effects of any kind. In order to design new mortars for restoration applications, the understanding of the function of the old, authentic mortars is prerequisite [8, 28]. The information gained by the microstructure analysis is exploited, in order to achieve similar function in terms of water restrain, porosity, and strength. Producing and testing new mortars in laboratory scale is a necessary step, in order to give guidelines to the field but also in order to test the availability in the market materials. The microstructure analysis of repair mortars is performed mainly for research reasons and rarely as a routine work. It is a safe way, though, to record the performance of the tested materials in a microscale. The utilization of SEM for characterization of raw materials used in mortars in order to improve certain properties, such as hydrophobicity, thermal properties, strength, and durability, is usually the first step towards their characterization [29, 30]. This is of a special value when researching new materials and products which are incorporated in mortar structure (Figure 7). The pros and cons can be recorded in relation to the amount, the curing regime, and the combination of the added materials.

**Figure 8:**
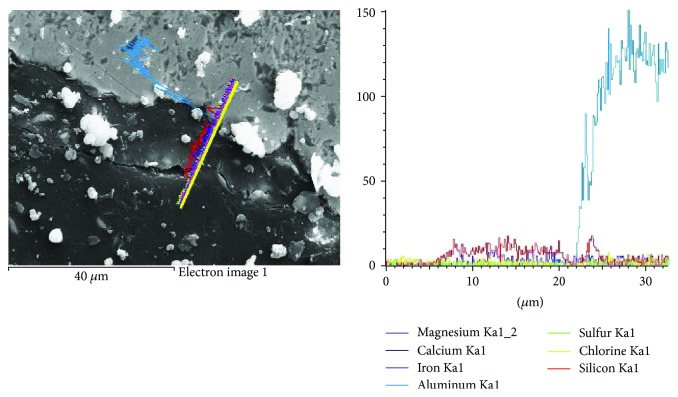
X-ray line scanning across the contact zone in mortar structure.

The energy dispersive X-ray *line scanning* performed across the contact zone between different materials is also a powerful tool explaining the material's behavior in terms of strength and porosity (Figure 8).

**Figure 9:**
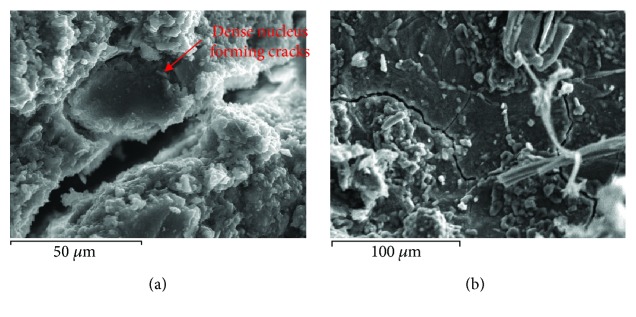
(a) Excess of nanoparticles in pastes forming cracking tendency. (b) Water-repellant layer on mortar which has been cracked.

Modern approaches in order to produce “smart” mortars which retain their traditional character but exploit new technologies for improving inherent weaknesses are of great interest in the last decades. Nanotechnology, for example, gave important products which have been successfully introduced in many traditional applications and improved mortar performance [31, 32]. The type and percentage of nanoparticles have been studied in detail by SEM, and valuable information have been revealed. For example, a high amount of nanoparticles (>5 wt % of binder) was decided that it had to be avoided in traditional systems, as SEM revealed cracking tendency and bad cohesion to the binder (Figure 9(a)). On the other hand, efforts to apply coatings in order to render mortars water-repellant can be recorded and assist towards the decision on how successful the intervention was, in terms of microstructure, or even better, the technique, assist in understanding the reason of failure, when that was the case (Figure 9(b)).

**Figure 10:**
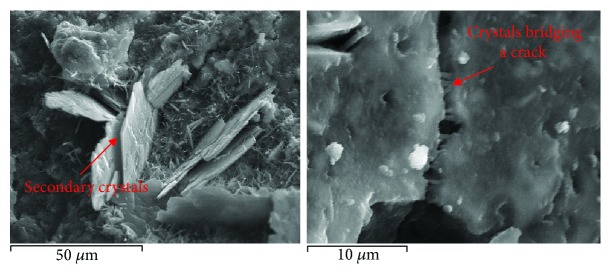
Self-healing approach by the formation of secondary products in pores and cracks of mortars.

Also, the role of additives in self-healing properties can be uniquely detected and identified and analyzed as healing products. That means that the morphology, place of growth, and stoichiometry of the formed products can be revealed, and the effectiveness of the healing process can be estimated [33] (Figure 10).

## 4. Conclusions

The detrimental role of microstructure in the macroscopic properties of building materials and in mortars, in particular, is well known. To fully benefit from microstructure control, it is important to properly apply the full range of available microscopic techniques. Microscopes have been proven valuable tools to researchers, in order to detect damages, identify phases, and develop improved materials used in the construction field. A number of analytical techniques are needed to be combined for the complete characterization of historic mortar; scanning electron microscopy highlights the diversity of microstructures through direct observation and microanalysis as well. The possibility of penetration into the structure having a direct observation in the scale of *μ*m or nm permits the detection of inherent defects, as well as ageing or deterioration effects, which influence the material's behavior. The pieces of information taken contribute to mortar quality estimation, by quantifying the mass density, topography of pores and cracks, distribution of aggregates, and additions in the binder and density of binder-aggregate interfaces. The ability of penetrating deeper and having a direct observation of the structure, in the scale of micro or nano, allows detection of inherent defects, as well as ageing or deterioration effects, which determine the material's macroscopic behavior. SEM is an irreplaceable tool in order to understand the structure of mortars, both through image observation and also through the provided analysis. The study of building materials has benefited greatly from SEM and that in an extent explains the long-lasting use of it in material research and the need to further evolve the technique. The understanding of old mortars and the evolution of building materials, in order to cover different construction needs, was in a significant extent based on it. In this way, compatibility with the authentic structures could be achieved, but also new technologies could be tested, in order to assist towards mortar's durability. The latter seems crucial in order to produce innovative traditional mortars, preserving the principles of compatibility with increased durability. Through SEM possibilities, the reveal of ancient mortars was possible and the puzzle of missing information, explaining the behavior of the materials, was enlightened. Working directly on images and at the same time having quantitative data on the composition were a revolutionary practice that was widely applied for producing high-quality mortars that can be safely used for repair works and also plan the future for high-performance materials.

## Data Availability

The data used to support the findings of this study are included within the article.
